# Association of Homocysteine with Arterial Stiffness and Kidney Injury Biomarkers in Patients with Suspected Coronary Artery Disease

**DOI:** 10.3390/jcm15134961

**Published:** 2026-06-25

**Authors:** Nejc Piko, Sebastjan Bevc, Franjo Husam Naji, Robert Ekart

**Affiliations:** 1 Department of Dialysis, University Medical Centre Maribor, 2000 Maribor, Slovenia; robert.ekart2@guest.arnes.si; 2 Department of Nephrology, University Medical Centre Maribor, 2000 Maribor, Slovenia; sebastjan.bevc@gmail.com; 3 Medical Faculty, University of Maribor, 2000 Maribor, Slovenia; 4 Clinical Department of Cardiology and Angiology, University Medical Centre Maribor, 2000 Maribor, Slovenia; franjo.naji@yahoo.com

**Keywords:** homocysteine, arterial stiffness, carotid–femoral pulse wave velocity, chronic kidney disease, cardiorenal syndrome, vascular remodeling

## Abstract

**Background**: Hyperhomocysteinemia (homocysteine [Hcy] ≥15 μmol/L) is frequently observed in patients with impaired kidney function and has been associated with vascular remodeling and increased cardiovascular risk. We aimed to evaluate the relationship between Hcy, arterial stiffness, coronary artery disease (CAD), peripheral arterial disease, and biomarkers of kidney injury in patients undergoing elective coronary angiography. **Methods**: In this prospective observational study, 133 patients undergoing elective coronary angiography were stratified according to serum Hcy levels (Hcy <15 vs. Hcy ≥15 μmol/L). CAD severity was assessed angiographically. Arterial stiffness was evaluated using carotid–femoral pulse wave velocity (cfPWV), while peripheral arterial disease was assessed using ankle–brachial index (ABI). Kidney function was evaluated using serum creatinine, estimated glomerular filtration rate (eGFR), cystatin C, and urinary albumin-to-creatinine ratio (UACR). Correlation, multivariable regression, logistic regression, and receiver operating characteristic (ROC) analyses were performed. **Results**: Patients with hyperhomocysteinemia demonstrated significantly worse kidney function, including higher serum creatinine, cystatin C, and UACR levels, and lower eGFR (all *p* < 0.01). Patients with elevated Hcy levels also exhibited significantly higher cfPWV values (11.4 ± 3.3 vs. 9.7 ± 2.1 m/s, *p* < 0.001). Hcy correlated positively with cystatin C, creatinine, UACR, and cfPWV, and inversely with eGFR. In multivariable linear regression analysis, Hcy remained independently associated with increased cfPWV after adjustment for age, sex, and eGFR (β = 0.137, 95% CI 0.047–0.226, and *p* = 0.003). This association remained significant in sensitivity analyses incorporating hypertension, diabetes mellitus, LDL cholesterol, and statin therapy (β = 0.124, 95% CI 0.032–0.216, and *p* = 0.008). No independent associations were observed between Hcy and angiographic CAD severity or ABI values. ROC analysis demonstrated modest discrimination for elevated arterial stiffness (AUC = 0.66, 95% CI 0.56–0.76) and good discrimination for impaired kidney function (AUC = 0.82, 95% CI 0.69–0.92). **Conclusions**: Elevated Hcy levels were independently associated with impaired kidney function and increased central arterial stiffness, but not with angiographic CAD severity or peripheral arterial disease. These findings suggest that hyperhomocysteinemia may reflect cardiorenal vascular dysfunction and diffuse vascular remodeling rather than focal obstructive atherosclerotic disease. Further studies are needed to determine its clinical utility and prognostic value.

**What was known:**

1. Hyperhomocysteinemia is frequently observed in patients with chronic kidney disease and has been associated with endothelial dysfunction, oxidative stress, arterial stiffness, and increased cardiovascular risk. However, its relationship with central arterial stiffness and kidney injury biomarkers remains incompletely understood.
2. Previous studies demonstrated associations between homocysteine and cardiovascular disease, but data simultaneously evaluating homocysteine, arterial stiffness, kidney dysfunction, peripheral arterial disease, and angiographically confirmed coronary artery disease within the same patient population are limited.
3. It remains unclear whether homocysteine primarily reflects obstructive atherosclerotic coronary disease or diffuse vascular remodeling and cardiorenal vascular dysfunction. Therefore, further studies integrating vascular and kidney biomarkers are needed.

**This study adds:**

1. Elevated homocysteine levels were independently associated with impaired kidney function and increased central arterial stiffness, but not with angiographic coronary artery disease severity or peripheral arterial disease.
2. Our findings suggest that hyperhomocysteinemia may primarily reflect diffuse vascular remodeling, vascular aging, and cardiorenal vascular dysfunction rather than advanced focal obstructive atherosclerotic disease.
3. Homocysteine may represent a useful additional biomarker for identifying patients with early vascular dysfunction, increased arterial stiffness, and increased cardiorenal risk beyond traditional cardiovascular risk assessment.

**Potential impact:**

1. Homocysteine measurement may help identify patients with early cardiorenal vascular dysfunction and increased central arterial stiffness, even in the absence of advanced obstructive coronary artery disease.
2. Assessment of homocysteine together with kidney injury biomarkers and arterial stiffness parameters may improve cardiovascular and renal risk stratification in patients with chronic kidney disease and high cardiovascular risk.
3. Our findings support a broader vascular interpretation of hyperhomocysteinemia and may encourage further research into its role in vascular remodeling, vascular aging, and cardiorenal prevention strategies.

## 1. Introduction

Homocysteine (Hcy) is a sulfur-containing amino acid generated during methionine metabolism and has emerged as an important biomarker of cardiovascular and renal disease [1,2]. Hyperhomocysteinemia, commonly defined as Hcy ≥ 15 μmol/L, is frequently observed in patients with impaired kidney function due to reduced renal clearance, altered methionine metabolism, uremic inhibition of key metabolic enzymes, and deficiencies of folate and vitamins B6 and B12 [3]. Beyond being a marker of kidney dysfunction, elevated Hcy has been implicated in endothelial dysfunction, oxidative stress, vascular smooth muscle proliferation, impaired nitric oxide bioavailability, inflammation, and vascular remodeling, all of which contribute to increased arterial stiffness and cardiovascular risk [4].

Cardiovascular disease remains the leading cause of morbidity and mortality in patients with chronic kidney disease (CKD), and non-traditional cardiovascular risk factors such as oxidative stress, endothelial dysfunction, chronic inflammation, and hyperhomocysteinemia are increasingly recognized as important contributors to this excess risk [5]. Previous studies have demonstrated associations between elevated Hcy levels and CKD progression, albuminuria, endothelial dysfunction, arterial stiffness, and adverse cardiovascular outcomes. However, the pathophysiological role of Hcy in cardiorenal disease remains incompletely understood, particularly regarding its relationship with central arterial stiffness, kidney injury biomarkers, and angiographically confirmed coronary artery disease (CAD) within the same patient population.

Emerging evidence suggests that Hcy may be more closely associated with early vascular dysfunction and arterial remodeling than with the severity of obstructive epicardial CAD. Increased arterial stiffness, commonly assessed by carotid–femoral pulse wave velocity (cfPWV), represents an established marker of vascular ageing and an independent predictor of cardiovascular morbidity and mortality. Similarly, biomarkers such as cystatin C and albuminuria provide sensitive indicators of early kidney injury and cardiovascular risk beyond what the estimated glomerular filtration rate (eGFR) alone can provide. Nevertheless, comprehensive studies simultaneously evaluating Hcy, arterial stiffness, kidney dysfunction, and coronary atherosclerotic burden remain limited.

Furthermore, conflicting results regarding the cardiovascular benefits of Hcy-lowering therapy with vitamin supplementation have renewed interest in clarifying the clinical and pathophysiological significance of hyperhomocysteinemia in cardiorenal vascular disease [6]. A better understanding of these associations may improve cardiovascular and renal risk stratification in patients with suspected CAD.

Therefore, the aim of our study was to evaluate the association between Hcy, arterial stiffness, CAD, peripheral arterial disease, and multiple biomarkers of kidney injury, including eGFR, cystatin C, and urinary albumin-to-creatinine ratio (UACR), in patients undergoing elective coronary angiography.

## 2. Methods

### 2.1. Study Population

This prospective observational study included 133 consecutive patients admitted to the Department of Cardiology and Angiology, Clinic for Internal Medicine, University Medical Centre Maribor, Slovenia, between 1 January 2018 and 1 February 2020, for elective diagnostic coronary angiography. Indications for coronary angiography included previously positive non-invasive testing for myocardial ischemia, including exercise stress testing or myocardial perfusion scintigraphy. None of the included patients had acute coronary syndrome at the time of enrollment.

All participants were aged ≥ 18 years and provided written informed consent prior to study inclusion. Exclusion criteria included atrial fibrillation, significant aortic stenosis, pregnancy, active malignancy, and age below 18 years. Patients with atrial fibrillation and aortic stenosis were excluded because these conditions may significantly interfere with pulse wave analysis and arterial stiffness measurements.

Baseline demographic characteristics, cardiovascular risk factors, comorbidities, and prescribed medications were recorded at enrollment. Body mass index (BMI) was calculated as body weight divided by height squared (kg/m^2^). Peripheral venous blood samples were obtained before coronary angiography. Laboratory analyses included serum hemoglobin, creatinine, cystatin C, C-reactive protein (CRP), lipid profile, triglycerides, and N-terminal pro-B-type natriuretic peptide (NT-proBNP). UACR was assessed using a spot urine sample and expressed in g/mol. eGFR was calculated using the Chronic Kidney Disease Epidemiology Collaboration (CKD-EPI) 2009 creatinine equation. CKD was defined as eGFR < 60 mL/min/1.73 m^2^ (KDIGO G3–G5).

The study protocol was approved by the National Medical Ethics Committee of the Republic of Slovenia (approval number 0120–32/2017/4) and conducted in accordance with the Declaration of Helsinki and Good Clinical Practice guidelines.

### 2.2. Homocysteine Measurements

Serum Hcy concentrations were measured prior to coronary angiography using a chemiluminescent microparticle immunoassay (Dimension Vista^®^, Siemens Healthcare Diagnostics, Newark, DE, USA). Hyperhomocysteinemia was defined as Hcy ≥ 15 μmol/L. Patients were stratified into two groups according to Hcy levels: Hcy < 15 μmol/L and Hcy ≥ 15 μmol/L.

### 2.3. Atherosclerosis Risk Assessment

All patients underwent coronary angiography for assessment of obstructive CAD. CAD severity was categorized according to the number of major epicardial coronary arteries with significant stenosis as zero-vessel, one-vessel, two-vessel, or three-vessel disease. Microvascular coronary dysfunction was not specifically evaluated.

Peripheral arterial disease was assessed using ankle–brachial index (ABI) measurements obtained with a validated automated waveform analysis device (MESI^®^, Ljubljana, Slovenia). Systolic blood pressure was measured simultaneously at both ankles and the right brachial artery, and ABI was calculated automatically as the ratio of ankle to brachial systolic blood pressure. The mean ABI value of both lower extremities was used for statistical analysis.

Arterial stiffness measurements were performed before coronary angiography using applanation tonometry (SphygmoCor^®^, AtCor Medical, Sydney, Australia). All measurements were conducted between 10:00 AM and 12:00 PM after a 5–10 min resting period in the supine position under standardized environmental conditions.

Pulse wave analysis was performed at the radial artery. Measurements were accepted only if predefined quality criteria were met, including operator index ≥ 80%, pulse height variation ≤ 5%, and diastolic variation ≤ 5%. Pulse pressure (PP) was calculated as the difference between systolic and diastolic blood pressure. The augmentation index normalized to a heart rate of 75 beats/minute (AIx@75) was calculated as augmentation pressure divided by pulse pressure. Ejection duration (ED) and subendocardial viability ratio (SEVR) were additionally derived from pulse wave analysis.

cfPWV, considered the gold-standard measure of central arterial stiffness, was calculated as the distance between carotid and femoral recording sites divided by pulse transit time. Three technically acceptable cfPWV measurements were obtained for each patient, and the mean value was used for analysis.

### 2.4. Statistical Analysis

Statistical analyses were performed using SPSS version 28.0 (IBM Corp., Armonk, NY, USA), with supplementary regression and receiver operating characteristic (ROC) analyses conducted in Python (version 3.13). Continuous variables were expressed as mean ± standard deviation (SD) for normally distributed data or median and interquartile range (IQR) for non-normally distributed data, while categorical variables were presented as frequencies and percentages. Normality was assessed using the Shapiro–Wilk test.

Comparisons between patients with Hcy < 15 μmol/L and Hcy ≥ 15 μmol/L were performed using the independent-samples Student’s *t*-test or Mann—Whitney U test, as appropriate, for continuous variables, and the chi-square test for categorical variables. Associations between Hcy and vascular or renal parameters were evaluated using Spearman’s rank correlation coefficients.

Multivariable linear regression analysis was performed to evaluate the independent association between Hcy and central arterial stiffness, with cfPWV entered as the dependent variable. The primary model included age, sex, and eGFR as covariates. Additional sensitivity analyses were performed incorporating hypertension, diabetes mellitus, LDL cholesterol, and statin therapy to assess the robustness of the observed associations. Multivariable logistic regression analysis was used to identify predictors of elevated arterial stiffness, defined as cfPWV > 10 m/s.

ROC curve analysis was performed to assess the ability of serum Hcy levels to discriminate between patients with elevated arterial stiffness (cfPWV > 10 m/s) and impaired kidney function (eGFR < 60 mL/min/1.73 m^2^). Discriminatory performance was quantified using the area under the ROC curve (AUC) with corresponding 95% confidence intervals (CI). Optimal cut-off values were determined using the Youden index. All statistical tests were two-sided, and a *p*-value < 0.05 was considered statistically significant.

## 3. Results

A total of 133 patients were included in the final analysis. The mean age of the study population was 65.0 ± 9.2 years, and 63.2% of patients were male. The most prevalent comorbidities were arterial hypertension (n = 93, 69.9%), dyslipidemia (n = 67, 50.4%), diabetes mellitus (n = 24, 18.0%), heart failure (n = 12, 9.0%), and CKD (n = 10, 7.5%). The most commonly prescribed medications included acetylsalicylic acid (n = 102, 76.7%), statins (n = 89, 66.9%), beta-blockers (n = 82, 61.7%), angiotensin-converting enzyme inhibitors (n = 67, 50.4%), diuretics (n = 43, 32.3%), calcium channel blockers (n = 28, 21.1%), metformin (n = 21, 15.8%), angiotensin receptor blockers (n = 20, 15.0%), and alpha-blockers (n = 17, 12.8%).

**Figure 1:**
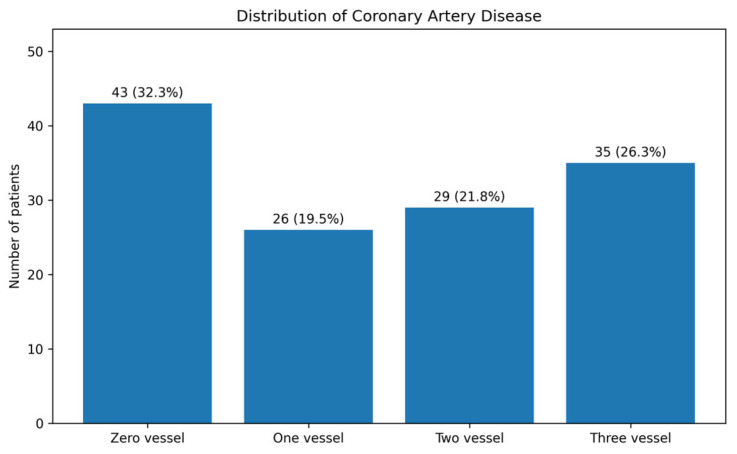
Distribution of coronary artery disease severity according to coronary angiography (n = 133). Values above the bars represent the absolute number of patients and corresponding percentage of the study population.

The mean eGFR of the study population was 75.5 ± 17.2 mL/min/1.73 m^2^, while the mean serum creatinine and cystatin C levels were 91.3 ± 65.4 μmol/L and 1.11 ± 0.71 mg/L, respectively. Mean cfPWV was 10.3 ± 2.7 m/s. Demographic data, laboratory values, arterial stiffness parameters, and mean ABI values are detailed in Table 1. The coronary angiography results and the CAD distribution among the included patients are shown in Figure 1.

Hyperhomocysteinemia (Hcy ≥ 15 μmol/L) was present in 28.6% of patients. Median serum Hcy level was 12.9 μmol/L (IQR 11.1–15.8). Patients with hyperhomocysteinemia were older and demonstrated worse kidney function, including higher serum creatinine, higher cystatin C, lower eGFR, and higher UACR values. Additionally, patients with elevated Hcy demonstrated significantly higher cfPWV values. Comparison of clinical and laboratory parameters is presented in Table 2. 

Spearman correlation analysis demonstrated significant associations between Hcy and multiple kidney function and vascular parameters. Serum Hcy showed a moderate inverse correlation with eGFR (r = −0.485, *p* < 0.001), while positive correlations were observed between Hcy and serum cystatin C (r = 0.608, *p* < 0.001), creatinine (r = 0.443, *p* < 0.001), and UACR (r = 0.311, *p* = 0.009). Furthermore, Hcy demonstrated a significant positive correlation with cfPWV (r = 0.328, *p* < 0.001), indicating an association between elevated Hcy levels and increased central arterial stiffness. No significant associations were observed between Hcy and AIx, AIx@75, PP, SEVR, or ABI values. Older age was also associated with higher Hcy levels (r = 0.328, *p* < 0.001). No independent association was observed between Hcy levels and angiographic severity of CAD.

Multivariable linear regression analysis was performed with cfPWV as the dependent variable and Hcy, age, sex, and eGFR entered as independent variables. The final multivariable regression model remained statistically significant and explained approximately one-third of the variability in cfPWV (adjusted R^2^ = 0.288, *p* < 0.001). After adjustment for age, sex, and eGFR, Hcy remained independently associated with higher cfPWV values (β = 0.137, 95% CI 0.047–0.226, *p* = 0.003). Age was also independently associated with higher cfPWV values (β = 0.121, 95% CI 0.075–0.167, *p* < 0.001), while sex and eGFR were not independently associated with arterial stiffness in the adjusted model.

To further evaluate potential confounding, additional multivariable models incorporating hypertension, diabetes mellitus, LDL cholesterol, and statin therapy were constructed. Hcy remained independently associated with higher cfPWV values after adjustment for these additional cardiovascular risk factors (β = 0.123, 95% CI 0.032–0.215, *p* = 0.009) and after further adjustment for statin therapy (β = 0.124, 95% CI 0.032–0.216, *p* = 0.008), confirming the robustness of the observed association.

Patients were additionally stratified according to elevated arterial stiffness (defined as cfPWV > 10 m/s). In multivariable logistic regression analysis, older age independently predicted elevated arterial stiffness (OR 1.13, 95% CI 1.07–1.20, *p* < 0.001). Although elevated Hcy levels were associated with higher cfPWV values in univariate analyses, this association did not remain statistically significant after multivariable logistic regression adjustment (OR 1.09, 95% CI 0.97–1.22, *p* = 0.165).

**Figure 2:**
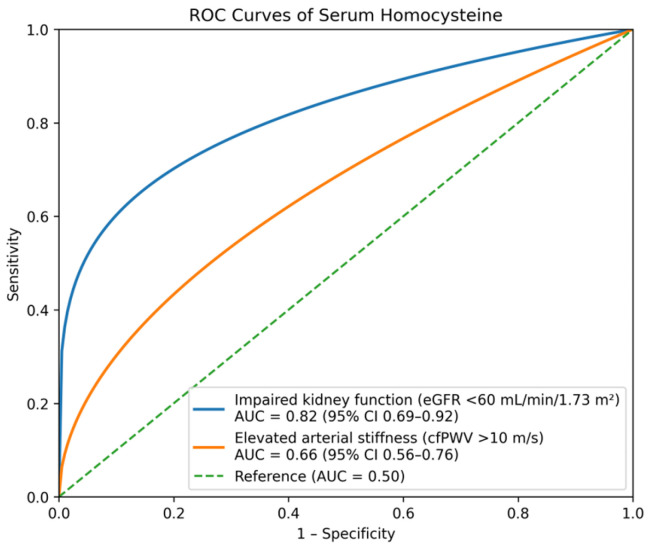
Discriminatory Performance of Homocysteine for Elevated Arterial Stiffness and Impaired Kidney Function.

ROC curve analysis was performed to evaluate the discriminatory performance of serum Hcy for identifying elevated arterial stiffness (cfPWV > 10 m/s) and impaired kidney function (eGFR < 60 mL/min/1.73 m^2^) (Figure 2). Hcy demonstrated modest discrimination for elevated arterial stiffness (AUC = 0.66, 95% CI 0.56–0.76). The optimal cut-off value was 12.3 μmol/L, corresponding to a sensitivity of 73.1% and a specificity of 58.2%. In contrast, Hcy demonstrated good discrimination for impaired kidney function (AUC = 0.82, 95% CI 0.69–0.92). The optimal cut-off value was 14.6 μmol/L, yielding a sensitivity of 81.0% and a specificity of 73.6%. These findings suggest that Hcy is more strongly associated with impaired kidney function than with elevated arterial stiffness.

## 4. Discussion

In this prospective observational study of patients undergoing elective coronary angiography, elevated Hcy levels were independently associated with impaired kidney function and increased central arterial stiffness. Higher Hcy concentrations correlated with lower eGFR and higher serum creatinine, cystatin C, albuminuria, and cfPWV values. Importantly, the association between Hcy and cfPWV remained significant after adjustment for age, sex, kidney function, and additional cardiovascular risk factors, supporting an independent relationship between hyperhomocysteinemia and vascular remodeling. However, although Hcy was significantly associated with arterial stiffness, its ability to discriminate patients with elevated cfPWV was modest (AUC = 0.64), suggesting limited utility as a standalone predictive marker. Instead, Hcy may be better regarded as a complementary biomarker reflecting underlying vascular remodeling and cardiorenal dysfunction rather than a direct predictor of arterial stiffness. Whether Hcy provides incremental prognostic or predictive value beyond established cardiovascular risk factors remains to be determined. In contrast, no independent associations were observed between Hcy and either angiographic CAD severity or peripheral arterial disease, supporting the concept that hyperhomocysteinemia may be more closely linked to diffuse vascular dysfunction and arterial remodeling than to focal obstructive atherosclerotic disease.

The strong association between Hcy and kidney dysfunction observed in our study is consistent with previous reports demonstrating progressive increases in Hcy levels with declining renal function. Hyperhomocysteinemia is common in patients with CKD and becomes highly prevalent in advanced stages of kidney disease [7,8,9,10,11]. In a large population-based cohort, Francis et al. reported strong associations between elevated Hcy levels, reduced eGFR, and increased albuminuria, with individuals exhibiting eGFR <60 mL/min/1.73 m^2^ having an approximately tenfold higher likelihood of hyperhomocysteinemia [12]. Similar findings have been reported in diabetic populations [13], while He et al. demonstrated that elevated Hcy and albuminuria were independently associated with increased cerebrovascular risk among hypertensive patients [14]. Collectively, these findings support the concept that Hcy reflects early cardiorenal dysfunction, even in individuals with relatively preserved kidney function.

Particularly noteworthy were the significant associations between Hcy, cystatin C, albuminuria, and eGFR. While serum creatinine and eGFR remain the standard measures of renal function, cystatin C and albuminuria are increasingly recognized as sensitive markers of early kidney injury and cardiovascular risk. Compared with creatinine-based estimates, cystatin C is less influenced by muscle mass and may detect renal dysfunction at an earlier stage [15,16,17]. Data on the relationship between Hcy and cystatin C remain limited; however, both biomarkers have been linked to inflammation, oxidative stress, endothelial dysfunction, and adverse cardiovascular outcomes [18]. Dzielinska et al. reported independent associations between cystatin C, Hcy, and CAD severity in hypertensive patients [19], while elevated levels of both biomarkers have also been associated with ventricular remodeling and adverse outcomes in heart failure [20]. Our findings extend these observations by demonstrating a strong correlation between Hcy and cystatin C in patients undergoing coronary angiography, further supporting the role of Hcy as a marker of cardiorenal vascular dysfunction.

The relationship between Hcy and kidney dysfunction is likely complex and bidirectional. Declining renal function may promote hyperhomocysteinemia through impaired renal clearance, altered amino acid metabolism, chronic inflammation, oxidative stress, and deficiencies of folate and vitamins B6 and B12, all of which are important determinants of Hcy metabolism [2,6,21]. In parallel, albuminuria reflects endothelial dysfunction and systemic vascular injury even in the presence of preserved eGFR [22,23]. Conversely, experimental and clinical studies have implicated elevated Hcy levels in pathways associated with kidney injury and CKD progression, including oxidative stress, endothelial dysfunction, impaired nitric oxide bioavailability, mesangial proliferation, and microvascular damage [24,25,26]. Consistent with this concept, Jager et al. demonstrated that hyperhomocysteinemia independently predicted the development of albuminuria after adjustment for kidney function [25]. Nevertheless, given the observational nature of the available evidence, it remains uncertain whether Hcy acts as a causal mediator or primarily reflects underlying vascular and renal dysfunction.

One of the most important findings of our study was the independent association between Hcy and central arterial stiffness, assessed by cfPWV. Although Hcy has traditionally been regarded as an atherogenic molecule [27], accumulating evidence suggests that its vascular effects may be more closely related to diffuse vascular dysfunction, endothelial injury, vascular remodeling, and accelerated vascular aging than to focal obstructive epicardial CAD itself. In our study, Hcy demonstrated a significant positive association with cfPWV, and this relationship persisted even after adjustment for age, sex, and kidney function parameters. Importantly, the association remained significant in additional sensitivity analyses incorporating hypertension, diabetes mellitus, LDL cholesterol, and statin therapy, supporting the robustness of the observed relationship. Although Hcy remained independently associated with cfPWV in linear regression analyses, it did not independently predict elevated arterial stiffness defined as cfPWV >10 m/s in logistic regression models. This finding is not necessarily contradictory. Linear regression evaluates cfPWV as a continuous variable and therefore captures the full spectrum of arterial stiffness values, whereas dichotomization at a predefined threshold results in loss of information and reduced statistical power. The present findings suggest that Hcy is associated with incremental increases in arterial stiffness across the continuum of cfPWV values but may have limited ability to discriminate between patients above and below a specific clinical cutoff.

Hcy was not independently associated with angiographic CAD severity or ABI values. This finding is particularly important because it suggests that hyperhomocysteinemia may predominantly reflect early functional and structural vascular alterations rather than advanced obstructive atherosclerotic disease. The observed association with cfPWV, but not ABI or angiographic CAD burden, further supports the hypothesis that Hcy is more closely linked to central arterial stiffening and diffuse vascular remodeling than to peripheral obstructive atherosclerosis or focal coronary stenosis [25,26,27]. Since cfPWV represents a marker of central elastic artery stiffness and vascular aging, our findings imply that elevated Hcy levels may identify patients with subclinical vascular dysfunction and increased cardiorenal vascular risk even before the development of advanced macrovascular disease. The absence of significant associations between Hcy and angiographic CAD severity or ABI should also be interpreted in the context of the study size. Although no independent relationships were identified, the relatively modest sample size may have limited the ability to detect weaker associations. Therefore, the possibility of a type II error cannot be excluded, particularly for outcomes with lower event rates or less pronounced effect sizes. Larger studies are needed to determine whether the observed null findings reflect a true absence of association or insufficient statistical power.

Several biological mechanisms have been proposed to explain the observed association between Hcy and increased central arterial stiffness. Hyperhomocysteinemia has been shown to promote oxidative stress, chronic vascular inflammation, vascular smooth muscle cell proliferation, collagen accumulation, elastin degradation, and impaired endothelial nitric oxide bioavailability, all of which contribute to reduced arterial compliance and progressive vascular stiffening [28]. In addition to its proatherogenic properties, Hcy appears to exert important effects on vascular structure and function through diffuse endothelial injury and adverse vascular remodeling, thereby contributing to accelerated vascular aging and altered central hemodynamics. Previous studies support these observations. Zhang et al. demonstrated significant associations between Hcy and both cfPWV and carotid-radial pulse wave velocity in an elderly community-based cohort [28], while additional studies confirmed the relationship between elevated Hcy levels and arterial stiffness in hypertensive and high cardiovascular risk populations [29]. Importantly, our findings extend these observations to patients undergoing elective coronary angiography and demonstrate that the association between Hcy and central arterial stiffness persists even after adjustment for traditional cardiovascular risk factors and kidney function parameters. Taken together, these findings suggest that Hcy may be more strongly associated with diffuse vascular dysfunction and central arterial remodeling than with focal obstructive coronary atherosclerosis alone [29]. This distinction may partially explain why Hcy demonstrated a strong relationship with cfPWV, but not with angiographic CAD severity or ABI values, in our cohort.

The present study was conducted in a selected population of patients referred for elective coronary angiography following positive non-invasive ischemia testing. As a result, the cohort represented individuals with a relatively high baseline cardiovascular risk and a greater prevalence of cardiovascular risk factors, vascular abnormalities, and kidney dysfunction than would be expected in the general population. This should be considered when interpreting the observed associations and may partly explain the relatively high prevalence of hyperhomocysteinemia and arterial stiffness in our study population. Consequently, the applicability of our findings to asymptomatic or lower-risk populations remains uncertain.

Interestingly, Hcy was associated with cfPWV but not with AIx@75 or SEVR. Although these parameters also reflect vascular function and central hemodynamics, they are influenced by multiple physiological determinants, including heart rate, peripheral wave reflections, vascular tone, and ventricular performance [7,8,9]. In contrast, cfPWV is considered a more direct measure of large-artery stiffness and structural vascular remodeling [7]. The observed findings may therefore suggest that Hcy is more closely related to arterial wall remodeling and vascular aging than to functional hemodynamic alterations. Further studies are needed to clarify the relationship between Hcy and other indices of vascular function.

The lack of association between Hcy and angiographic CAD severity in our study deserves further consideration. First, our population consisted exclusively of patients referred for elective coronary angiography after positive non-invasive ischemia testing, resulting in a relatively selected cohort with high baseline cardiovascular risk. Second, patients with acute coronary syndromes, atrial fibrillation, and severe valvular disease were excluded, potentially limiting the inclusion of patients with more advanced cardiovascular pathology [28,29]. Third, coronary angiography primarily evaluates epicardial coronary stenosis and does not assess coronary microvascular dysfunction, endothelial dysfunction, or diffuse vascular remodeling. Consequently, the absence of an association between Hcy and angiographic CAD severity should be interpreted cautiously. In addition, CAD severity was categorized according to the number of major epicardial vessels with significant stenosis, a widely used but relatively simplified measure that does not capture lesion complexity, plaque characteristics, diffuse atherosclerotic burden, or coronary microvascular dysfunction. Patients with non-obstructive coronary arteries may still exhibit significant microvascular disease or diffuse atherosclerosis, both of which have been implicated in myocardial ischemia and adverse cardiovascular outcomes. Furthermore, advanced functional and imaging assessments, such as coronary flow reserve measurements, positron emission tomography, cardiac magnetic resonance perfusion imaging, or invasive coronary function testing, were not performed. Therefore, the overall burden of coronary vascular pathology may have been underestimated, potentially contributing to the lack of an observed association between Hcy and angiographic CAD severity. Given the established associations between Hcy, endothelial dysfunction, and vascular remodeling, future studies incorporating more comprehensive assessment of coronary atherosclerotic burden and microvascular function may provide additional insights into the relationship between hyperhomocysteinemia and coronary vascular disease [30,31].

Similarly, we did not observe a significant relationship between Hcy and ABI. Most patients had preserved ABI values, and only a minority had clinically established peripheral arterial disease, which may partially explain this finding. Furthermore, ABI primarily detects hemodynamically significant large-vessel peripheral arterial disease and may be less sensitive for identifying early-stage atherosclerosis, diffuse vascular remodeling, or microvascular dysfunction. Consequently, patients with subclinical peripheral vascular disease may have been classified as having normal peripheral circulation despite underlying vascular abnormalities. Additional modalities, including duplex ultrasonography, peripheral pulse wave velocity assessment, vascular imaging, or endothelial function testing, may provide a more comprehensive characterization of peripheral vascular disease and could potentially reveal associations with Hcy that were not detected using ABI alone [32]. Previous studies demonstrating associations between Hcy and peripheral arterial disease often included populations with more advanced vascular disease and higher prevalence of abnormal ABI values [32,33].

Although elevated Hcy levels were associated with impaired kidney function and increased arterial stiffness, the clinical implications of these findings remain uncertain. Given the observational design, it cannot be determined whether Hcy represents a modifiable risk factor, a causal mediator, or simply a marker of underlying cardiorenal dysfunction. Furthermore, previous interventional studies evaluating Hcy-lowering strategies have yielded inconsistent results. Therefore, prospective studies and randomized clinical trials are needed to determine whether Hcy provides incremental value for risk stratification and whether lowering Hcy can improve vascular or renal outcomes [33].

Our study has several limitations. First, the relatively modest sample size may have limited statistical power, particularly for analyses evaluating CAD severity and ABI. Consequently, the absence of significant associations for these outcomes should be interpreted cautiously, as weaker relationships may have remained undetected. Second, the study included only Caucasian patients from a single tertiary referral center in Slovenia and represented a selected high-risk population referred for elective coronary angiography following positive non-invasive ischemia testing. Therefore, the generalizability of our findings to other ethnic groups, healthcare settings, and lower-risk populations may be limited.

Third, the observational cross-sectional design precludes causal inference regarding the relationship between Hcy, kidney dysfunction, and arterial stiffness. Additionally, all biomarkers were assessed at a single time point, limiting evaluation of temporal changes and long-term variability. Future longitudinal studies with serial measurements are needed to clarify these relationships.

Fourth, residual confounding cannot be excluded. We did not assess vitamin B12, vitamin B6, folate status, inflammatory burden, dietary factors, smoking status, physical activity, medication adherence, socioeconomic factors, or genetic determinants of Hcy metabolism, including MTHFR polymorphisms, all of which may influence circulating Hcy concentrations. These factors may influence both Hcy concentrations and vascular function and could have contributed to the observed associations. Consequently, although the association between Hcy and cfPWV remained statistically significant after adjustment for measured covariates, residual confounding cannot be excluded. Therefore, the observed relationship should be interpreted as hypothesis-generating and requires confirmation in larger prospective studies with more comprehensive assessment of potential confounders.

Finally, coronary microvascular dysfunction was not specifically evaluated, CAD severity was assessed using a simplified vessel-based classification, and peripheral arterial disease assessment relied solely on ABI measurements. In addition, novel biomarkers of tubular kidney injury, such as NGAL, KIM-1, and DKK-3, were not assessed, and UACR was measured from a single spot urine sample. These methodological considerations may have limited the detection of more subtle associations between Hcy, vascular dysfunction, and kidney injury.

Despite these limitations, our study provides a comprehensive simultaneous evaluation of Hcy, kidney biomarkers, arterial stiffness, and angiographic CAD within the same patient population. The independent association between Hcy and central arterial stiffness, together with its strong relationship with kidney dysfunction markers, supports its potential role as an integrated biomarker of cardiorenal vascular dysfunction.

## 5. Conclusions

Elevated Hcy levels were independently associated with impaired kidney function and increased central arterial stiffness in patients undergoing elective coronary angiography. Hcy correlated with multiple biomarkers of kidney injury and remained independently associated with cfPWV after adjustment for age, sex, kidney function, and cardiovascular risk factors. In contrast, no independent association was observed with angiographic CAD severity or peripheral arterial disease. These findings suggest that hyperhomocysteinemia may primarily reflect diffuse vascular remodeling and cardiorenal vascular dysfunction rather than focal obstructive atherosclerotic disease. Further studies are needed to clarify its prognostic significance and potential clinical utility in cardiovascular and renal risk stratification.

## Figures and Tables

**Table 1:** Baseline demographic, laboratory, kidney, and vascular characteristics of the study population (n = 133).

Variable	Value
Demographic and anthropometric parameters	
Age, years	65.0 ± 9.2
BMI, kg/m^2^	28.3 ± 4.4
Laboratory parameters	
Hemoglobin, g/L	139.4 ± 13.2
CRP, mg/L	3.0 [3.0–3.0]
Total cholesterol, mmol/L	4.6 ± 1.1
LDL cholesterol, mmol/L	2.8 ± 1.0
HDL cholesterol, mmol/L	1.3 ± 0.4
Triglycerides, mmol/L	1.6 [1.2–2.3]
Kidney function parameters	
Creatinine, μmol/L	91.3 ± 65.4
eGFR, mL/min/1.73 m^2^	75.5 ± 17.2
Cystatin C, mg/L	1.11 ± 0.71
UACR, g/mol	12.0 [6.0–34.0]
Homocysteine, μmol/L	12.9 [11.1–15.8]
Vascular parameters	
ABI	1.0 ± 0.1
SEVR, %	166.0 ± 32.8
cfPWV, m/s	10.3 ± 2.7
AIx@75, %	25.5 ± 9.9
Pulse pressure, mmHg	47.2 ± 14.5

Data are presented as mean ± SD or median [IQR], as appropriate. BMI—body mass index; CRP—C-reactive protein; LDL—low-density lipoprotein; HDL—high-density lipoprotein; eGFR—estimated glomerular filtration rate; UACR—urinary albumin-to-creatinine ratio; ABI—ankle–brachial index; SEVR—subendocardial viability ratio; cfPWV—carotid–femoral pulse wave velocity; AIx—augmentation index.

**Table 2:** Comparison of clinical and laboratory parameters by homocysteine level.

Variable	Hcy < 15 (n = 81)	Hcy ≥ 15 (n = 52)	Mean Difference (95% CI)	*p*-Value ^a^
Age (years)	63.6 ± 9.3	68.5 ± 8.1	4.9 (1.7 to 8.1)	0.007 ^b^
Homocysteine (μmol/L)	11.5 ± 2.2	20.4 ± 6.5	8.9 (7.1 to 10.7)	<0.001 ^b^
Creatinine (μmol/L)	78.0 ± 16.5	124.3 ± 114.0	46.3 (10.2 to 82.4)	<0.001 ^c^
eGFR (mL/min/1.73 m^2^)	80.8 ± 11.6	62.1 ± 21.3	−18.7 (−25.4 to −12.0)	<0.001 ^b^
Cystatin C (mg/L)	0.9 ± 0.2	1.5 ± 1.2	0.6 (0.2 to 1.0)	<0.001 ^c^
cfPWV (m/s)	9.9 ± 2.2	11.4 ± 3.3	1.5 (0.5 to 2.5)	0.023 ^c^

Data are presented as mean ± SD. Mean difference (95% CI) represents the between-group difference (Hcy ≥ 15 minus Hcy < 15) with 95% confidence interval. Hcy, homocysteine; eGFR, estimated glomerular filtration rate; cfPWV, carotid-femoral pulse wave velocity. ^a^ *p*-value with corresponding statistical test used. ^b^ Student’s *t*-test. ^c^ Mann–Whitney U test.

## Data Availability

All data generated or analyzed during this study are included in this article. Further enquiries can be directed to the corresponding author.
